# Screening, identification and validation of CCND1 and PECAM1/CD31 for predicting prognosis in renal cell carcinoma patients

**DOI:** 10.18632/aging.102540

**Published:** 2019-12-18

**Authors:** Jian-Feng Yang, Shen-Nan Shi, Wen-Hao Xu, Yun-Hua Qiu, Jin-Zhou Zheng, Kui Yu, Xiao-Yun Song, Feng Li, Yu Wang, Rui Wang, Yuan-Yuan Qu, Hai-Liang Zhang, Xi-Qiu Zhou

**Affiliations:** 1 Department of Surgery, Pudong Branch of Longhua Hospital, Shanghai University of Traditional Chinese Medicine, Shanghai 200126, China; 2 Cancer Institute, Fudan University Shanghai Cancer Center, Shanghai 200032, P.R. China; 3 Department of Urology, Fudan University Shanghai Cancer Center, Shanghai 200032, P.R. China; 4 Department of Oncology, Shanghai Medical College, Fudan University, Shanghai 200032, P.R. China

**Keywords:** clear cell renal cell carcinoma, ccRCC, CCND1, PECAM1/CD31, biomarker

## Abstract

Clear cell renal cell carcinoma (ccRCC) is one of the most common cancers worldwide. Despite intense efforts to elucidate its pathogenesis, the molecular mechanisms and genetic characteristics of this cancer remain unknown. In this study, three expression profile data sets (GSE15641, GSE16441 and GSE66270) were integrated to identify candidate genes that could elucidate functional pathways in ccRCC. Expression data from 63 ccRCC tumors and 54 normal samples were pooled and analyzed. The GSE profiles shared 379 differentially expressed genes (DEGs), including 249 upregulated genes, and 130 downregulated genes. A protein-protein interaction network (PPI) was constructed and analyzed using STRING and Cytoscape. Functional and signaling pathways of the shared DEGs with significant p values were identified. Kaplan-Meier plots of integrated expression scores were used to analyze survival outcomes. These suggested that *FN1*, *ICAM1*, *CXCR4*, *TYROBP*, *EGF*, *CAV1*, *CCND1* and *PECAM1/CD31* were independent prognostic factors in ccRCC. Finally, to investigate early events in renal cancer, we screened for the hub genes *CCND1* and *PECAM1/CD31*. In summary, integrated bioinformatics analysis identified candidate DEGs and pathways in ccRCC that could improve our understanding of the causes and underlying molecular events of ccRCC. These candidate genes and pathways could be therapeutic targets for ccRCC.

## INTRODUCTION

Kidney cancer is one of the most common urinary tumors in the world, with an estimated 73,820 new cases and 14,770 deaths in the United States in 2019 [1]. The incidence and mortality of kidney cancer in China is also increasing. In 2015, the estimated number of new cases was 66,800 and the number of deaths was 23,400 [2]. Clear cell renal cell carcinoma (ccRCC) is the major subtype of kidney cancer and is the most common type of renal cell carcinoma (RCC) in adults. According to the World Health Organization, it is one of the most deadly urinary tumors with an annual global mortality rate of approximately 90,000 [3]. Although extensive research has been conducted on the mechanisms of carcinogenesis and progression, the etiology of ccRCC still remains unclear. The development and progression of RCC is reportedly associated with a variety of factors, including genetic aberrations and cellular or metabolic factors [4]. Considering the high morbidity and mortality of RCC, it is critical to reveal the causes and the underlying molecular mechanisms, and to explore molecular biomarkers for early diagnosis, prevention and personalized therapy.

Clear cell renal cell carcinoma represents a heterogeneous group of histologically similar neoplasms. Its development and progression are multistep processes characterized by aberrant genes, which subsequently lead to phenotypic cellular transformation [5]. RNA sequencing (RNA-Seq) has been used to detect genome-wide genetic changes [6]. Comprehensive and systematic study of the interactions between differentially expressed pathways and protein-coding genes can more accurately identify the biological changes that occur during the process of ccRCC carcinogenesis. Therefore, using these bioinformatics methods to analyze RNA-Seq data can aid the understanding of molecular pathogenesis and identify relevant tumor biomarkers. To fully comprehend the changes in gene expression that occur during ccRCC, RNA-Seq has been used to identify many key genes involved in disease progression. So far, the key drivers of carcinogenesis are still unknown, limiting the progress of ccRCC targeted therapy [7]. Therefore, understanding the pathogenesis of this disease remains a major challenge and many key genes are yet to be identified.

In this study, we first selected the gene sets GSE15641, GSE16441 and GSE66270 from the Gene Expression Omnibus (GEO). Second, we applied the R package ‘LIMMA’ from the Bioconductor project [8] and Venn diagram software to obtain the differentially expressed genes (DEGs) commonly found in the above three data sets. Third, the Database for Annotation, Visualization and Integrated Discovery (DAVID) was used to analyze these DEGs including molecular function (MF), cellular components (CC), biological processes (BP), and Kyoto Encyclopedia of Gene and Genome (KEGG) pathways. Fourth, we established a protein-protein interaction (PPI) network and then applied Cytotype MCODE (Molecular Complex Detection) for additional DEG analysis to identify some significant module. Fifth, cytoHubba was used to screen 10 hub genes. In addition, these hub genes were imported into the Kaplan Meier plotter online database to obtain important prognostic information (P < 0.05). Meanwhile, we further verified the expression of hub DEGs between ccRCC tissues and normal kidney tissues by gene expression profiling analysis (GEPIA; *P*<0.05). Finally, two DEGs (CCND1 and PECAM1/CD31) were generated. In summary, the aim of this study was to improve the understanding of the carcinogenesis of ccRCC by analyzing information about the genetic changes that occur during disease progression and by revealing the expression of biomarkers that may be used for clinical diagnosis, treatment, and disease progression monitoring.

## RESULTS

### Identification of DEGs in kidney cancer

**Figure 1:**
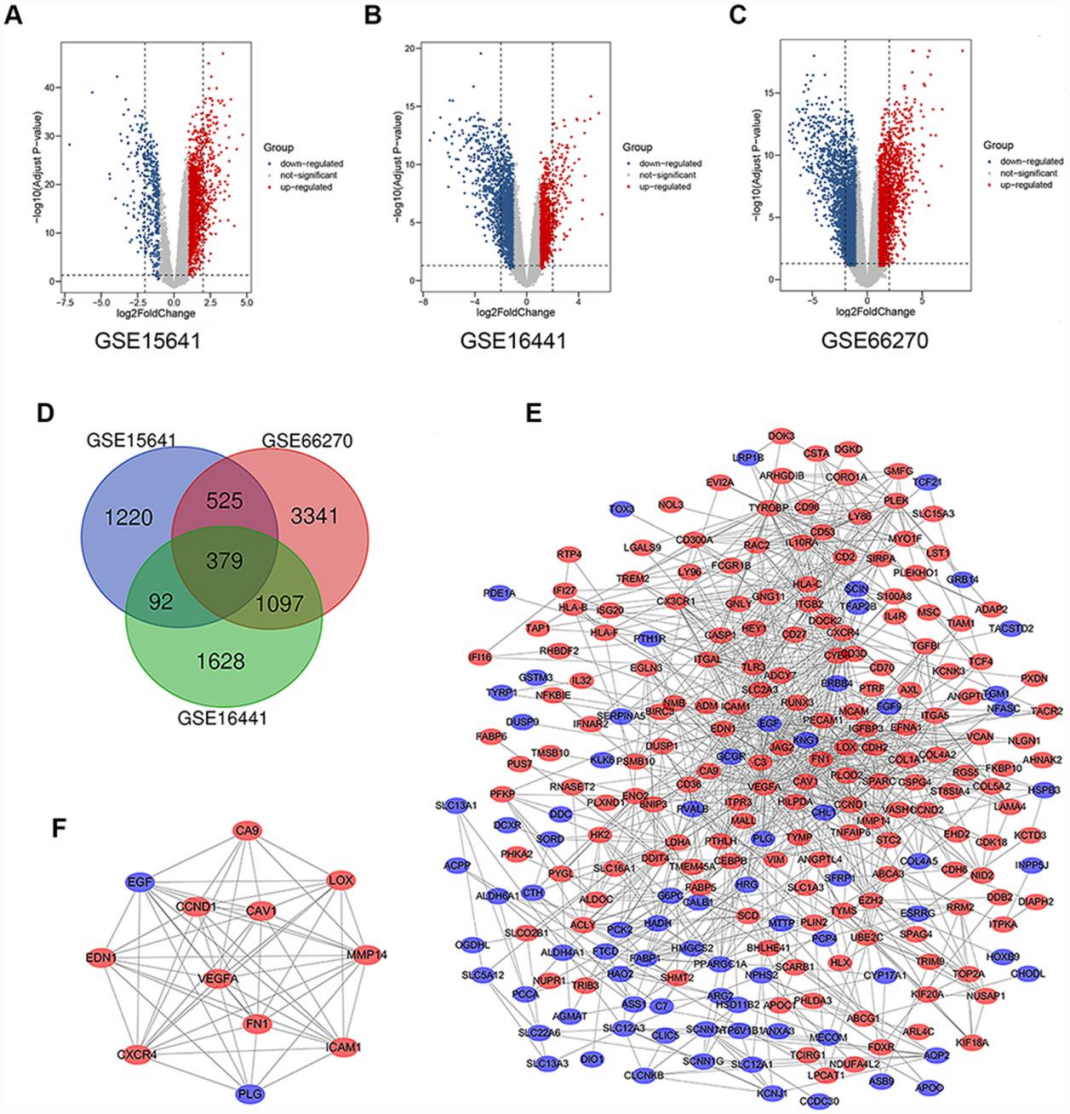
**Differential expression of data between two sets of samples, Venn diagram, PPI network and the most significant module of DEGs.** (**A**) GSE15641 data, (**B**) GSE16441 data, and (**C**) GSE66270 data. The red points represent upregulated genes screened on the basis of fold change > 1.0 and a corrected P-value of < 0.05. The blue points represent downregulation of the expression of genes screened on the basis of fold change < 1.0 and a corrected P-value of < 0.05. The black points represent genes with no significant difference. (**D**) DEGs were selected with a |fold change| >1 and P-value <0.05 among the mRNA expression profiling sets GSE15641, GSE16441 and GSE66270. The 3 datasets showed an overlap of 379 genes. (**E**) The PPI network of DEGs was constructed using Cytoscape. (**F**) The most significant module was obtained from PPI network with 12 nodes and 58 edges. Upregulated genes are marked in light red; downregulated genes are marked in light blue. Abbreviations: FC: fold change; GEO: Gene Expression Omnibus; DEGs: differentially expressed genes; PPI: protein–protein interaction.

There were 63 ccRCC tissues and 54 normal kidney tissues in our present study. Via limma software package, 2217, 3197 and 5343 DEGs from GSE15641, GSE16441 and GSE66270 were extracted respectively. The differential expression of multiple genes from two sets of sample data included in each of the three microarrays is shown in Figure 1A–1C. Then, Venn diagram software was used to identify the commonly DEGs in the three datasets. Results showed that a total of 379 commonly DEGs were detected, including 249 downregulated genes (logFC< 1) and 130 up-regulated genes (logFC> 1) in the ccRCC tissues (Figure 1D).

### DEGs gene ontology and KEGG pathway analysis in kidney cancers

To analyze the biological classification of DEGs, functional and pathway enrichment analyses were performed using DAVID. GO analysis results showed that changes in BP of DEGs were significantly enriched in angiogenesis, extracellular matrix organization, response to hypoxia, excretion and response to drug (Table 1). Changes in CC were mainly enriched in extracellular exosome, plasma membrane, extracellular space, cell surface, and integral component of plasma membrane (Table 1). Changes in MF of DEGs were mainly enriched in transporter activity, protein homodimerization activity, receptor binding, extracellular matrix structural constituent, and cysteine-type endopeptidase inhibitor activity involved in apoptotic process (Table 1). KEGG pathway analysis revealed that the DEGs were mainly enriched in viral myocarditis, Cell adhesion molecules, Phagosome, Biosynthesis of antibiotics and PI3K-Akt signaling pathway (Table 1).

**Table 1:** GO and KEGG pathway enrichment analysis of DEGs in ccRCC samples.

**Term**	**Description**	**Count in gene set**	**P-value**
Gene Ontology			
GO:0001525	Angiogenesis	24	4.59E-10
GO:0030198	Extracellular matrix organization	22	1.33E-09
GO:0001666	Response to hypoxia	20	4.94E-09
GO:0007588	Excretion	10	6.05E-08
GO:0042493	Response to drug	24	1.63E-07
GO:0070062	Extracellular exosome	128	2.66E-20
GO:0005886	Plasma membrane	142	4.29E-12
GO:0005615	Extracellular space	67	1.11E-11
GO:0009986	Cell surface	36	1.37E-09
GO:0005887	Integral component of plasma membrane	62	1.12E-08
GO:0005215	Transporter activity	19	2.19E-07
GO:0042803	Protein homodimerization activity	36	3.44E-06
GO:0005102	Receptor binding	21	4.90E-05
GO:0005201	Extracellular matrix structural constituent	9	6.94E-05
GO:0043027	Cysteine-type endopeptidase inhibitor activity involved in apoptotic process	6	9.09E-05
**Biological pathway**			
hsa05416	Viral myocarditis	12	6.03E-07
hsa04514	Cell adhesion molecules (CAMs)	17	3.51E-06
hsa04145	Phagosome	17	7.21E-06
hsa01130	Biosynthesis of antibiotics	20	1.29E-05
hsa04151	PI3K-Akt signaling pathway	26	2.30E-05
hsa04510	Focal adhesion	19	3.10E-05
hsa04066	HIF-1 signaling pathway	12	1.06E-04
hsa00010	Glycolysis / Gluconeogenesis	10	1.33E-04
hsa04512	ECM-receptor interaction	11	2.13E-04
hsa03320	PPAR signaling pathway	9	7.09E-04

### The significant modular analyses through DEGs protein–protein interaction network (PPI)

To identify the significant modular, the STRING online database (available online: http://string-db.org) and Cytoscape software were used to merge the 379 DEGs. The PPI network of DEGs was constructed (Figure 1E) and the most significant module was obtained using Cytoscape (Figure 1F). The functional analyses of genes involved in this module were analyzed using DAVID. GO analysis results showed that changes in BP of the significant module genes were significantly enriched in response to hypoxia, angiogenesis, positive regulation of peptidase activity, platelet degranulation and mammary gland alveolus development (Table 2). Changes in CC of the significant module genes were mainly enriched in platelet alpha granule lumen, extracellular space, extracellular region, cell surface, and cytoplasmic vesicle (Table 2). Changes in MF of the significant module genes were mainly enriched in peptidase activator activity, integrin binding and protein binding (Table 2). KEGG pathway analysis revealed that the significant module genes were mainly enriched in focal adhesion, bladder cancer, pathways in cancer, proteoglycans in cancer and viral myocarditis (Table 3).

**Table 2:** GO and KEGG pathway enrichment analysis of the significant module in ccRCC samples.

**Term**	**Description**	**Count in gene set**	**P-value**
Gene Ontology			
GO:0001666	Response to hypoxia	5	3.32E-06
GO:0001525	Angiogenesis	5	9.29E-06
GO:0010952	Positive regulation of peptidase activity	3	3.03E-05
GO:0002576	Platelet degranulation	4	3.57E-05
GO:0060749	Mammary gland alveolus development	3	5.28E-05
GO:0031093	Platelet alpha granule lumen	4	4.22E-06
GO:0005615	Extracellular space	7	5.39E-05
GO:0005576	Extracellular region	6	0.00157
GO:0009986	Cell surface	4	0.00361
GO:0031410	Cytoplasmic vesicle	3	0.00844
GO:0016504	Peptidase activator activity	3	1.73E-05
GO:0005178	Integrin binding	3	0.00203
GO:0005515	Protein binding	11	0.00843
GO:0001618	Virus receptor activity	2	0.04469
Biological pathway			
hsa04510	Focal adhesion	5	1.42E-04
hsa05219	Bladder cancer	3	0.00151
hsa05200	Pathways in cancer	5	0.00167
hsa05205	Proteoglycans in cancer	4	0.0025
hsa05416	Viral myocarditis	3	0.00291
hsa05212	Pancreatic cancer	3	0.00377
hsa04066	HIF-1 signaling pathway	3	0.00806
hsa04668	TNF signaling pathway	3	0.00994

**Table 3:** Functional roles of 10 hub genes.

**No.**	**Gene symbol**	**Full name**	**Function**
1	VEGFA	Vascular Endothelial Growth Factor A	Pathways: VEGF Signaling Pathway and Bladder cancer; GO: protein homodimerization activity and protein heterodimerization activity.
2	FN1	Fibronectin 1	Pathways: RET signaling and Cell surface interactions at the vascular wall; GO: heparin binding and protease binding.
3	ITGB1	Integrin Subunit Beta 2	Pathways: Activated TLR4 signalling and Focal Adhesion; GO: protein heterodimerization activity.
4	ICAM1	Intercellular Adhesion Molecule 1	Pathways: Interferon gamma signaling and Glucocorticoid receptor regulatory network.
5	CXCR4	C-X-C Motif Chemokine Receptor 4	Pathways: Human cytomegalovirus infection and Blood-Brain Barrier and Immune Cell Transmigration: VCAM-1/CD106 Signaling Pathways; GO: G protein-coupled receptor activity and ubiquitin protein ligase binding.
6	PECAM1	Platelet And Endothelial Cell Adhesion Molecule 1	Pathways: Blood-Brain Barrier and Immune Cell Transmigration: VCAM-1/CD106 Signaling Pathways and Innate Immune System
7	CCND1	Cyclin D1	Pathways: Gastric cancer and Bladder cancer; GO: protein kinase activity and enzyme binding.
8	TYROBP	TYRO Protein Tyrosine Kinase Binding Protein	Pathways: RET signaling and Innate Immune System; GO: identical protein binding and obsolete signal transducer activity, downstream of receptor.
9	EGF	Epidermal Growth Factor	Pathways: Gastric cancer and Vesicle-mediated transport; GO: calcium ion binding and epidermal growth factor receptor binding.
10	CAV1	Caveolin 1	Pathways: Focal Adhesion and TNF signaling (REACTOME); GO: identical protein binding and signaling receptor binding.

### Hub gene selection and survival outcomes of the cohorts

**Figure 2:**
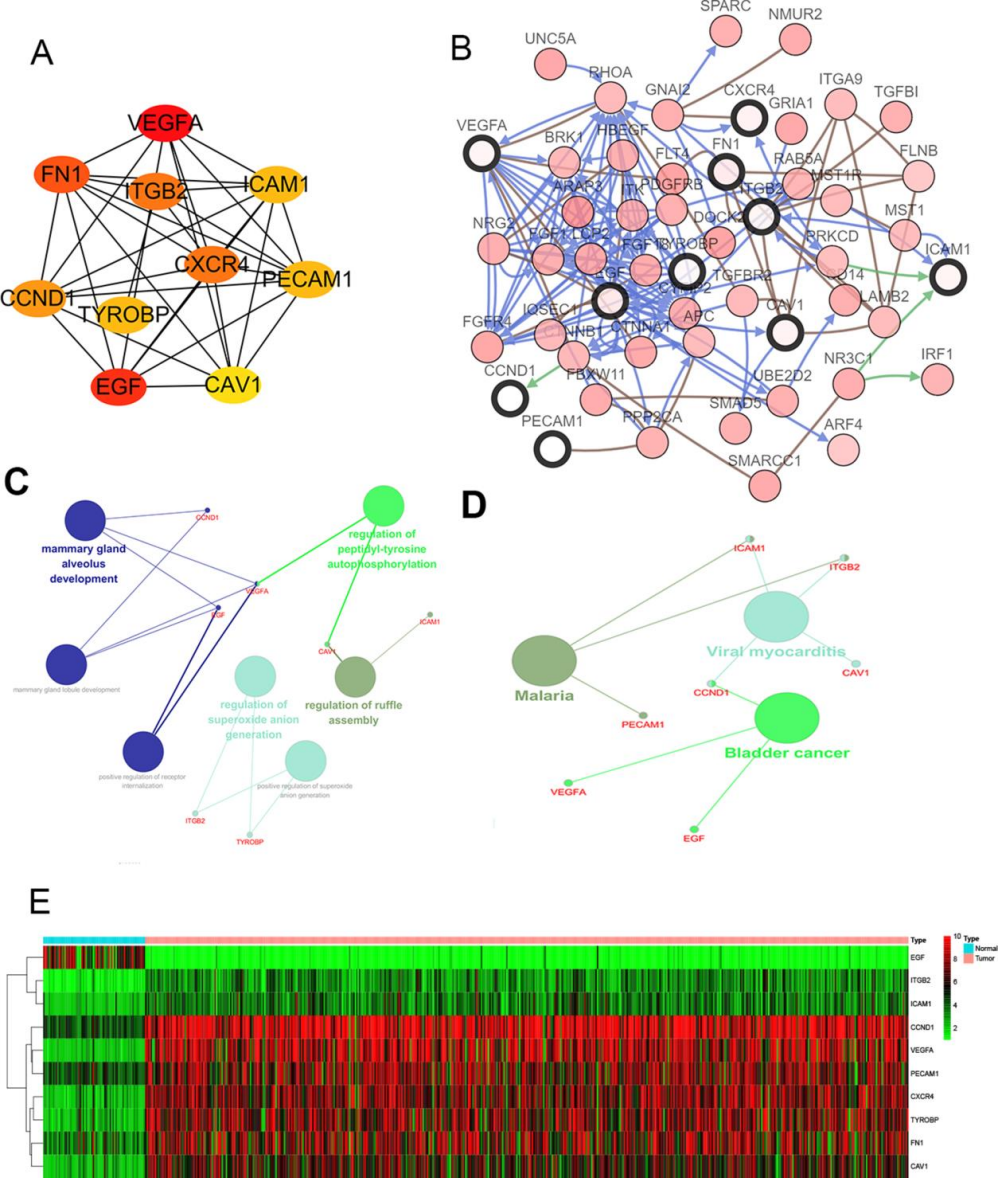
**Interaction network and analysis of the hub genes.** (**A**) Screen out the 10 most important hub genes using the cytoscape software plugin cytoHubba. (**B**) Hub genes and their co-expression genes were analyzed using cBioPortal. Nodes with bold black outline represent hub genes. Nodes with thin black outline represent the co-expression genes. (**C**) The biologic process functional annotation analysis of hub genes was performed by ClueGO and CluePedia. Different colors of nodes refer to the functional annotation of ontologies. Corrected P value <.01 was considered statistically significant. (**D**) The KEGG functional annotation analysis of hub genes was performed by ClueGO and CluePedia. Different colors of nodes refer to the functional annotation of ontologies. Corrected P value <.01 was considered statistically significant. (**E**) Hierarchical clustering heatmap of 10 most important hub genes was constructed depend on TCGA cohort. Red indicates that the expression of genes is relatively upregulated, green indicates that the expression of genes is relatively downregulated, and black indicates no significant changes in gene expression; gray indicates that the signal strength of genes was not high enough to be detected. Abbreviation: TCGA: the cancer genome atlas program; KEGG: Kyoto Encyclopedia of Genes and Genomes.

We filtered 30 hub genes that were identified by filtering according to the criterion of degrees >10 criteria (each node had more than 10 interactions), and the 10 most significant genes according to node degree were *VEGFA, FN1, ITGB2, ICAM1, CXCR4, PECAM1/CD31, TYROBP, CCND1, EGF, CAV1.* (Figure 2A) The names, abbreviations and functions for these hub genes are shown in Table 3. A network of the hub genes and their co-expression genes was analyzed using cBioPortal online platform (Figure 2B). The biological process and KEGG enrichment analysis of the hub genes is shown in Figure 2C–2D. Hierarchical clustering shows that the hub gene can basically distinguish between kidney cancer samples and non-cancer samples (Figure 2E).

**Figure 3:**
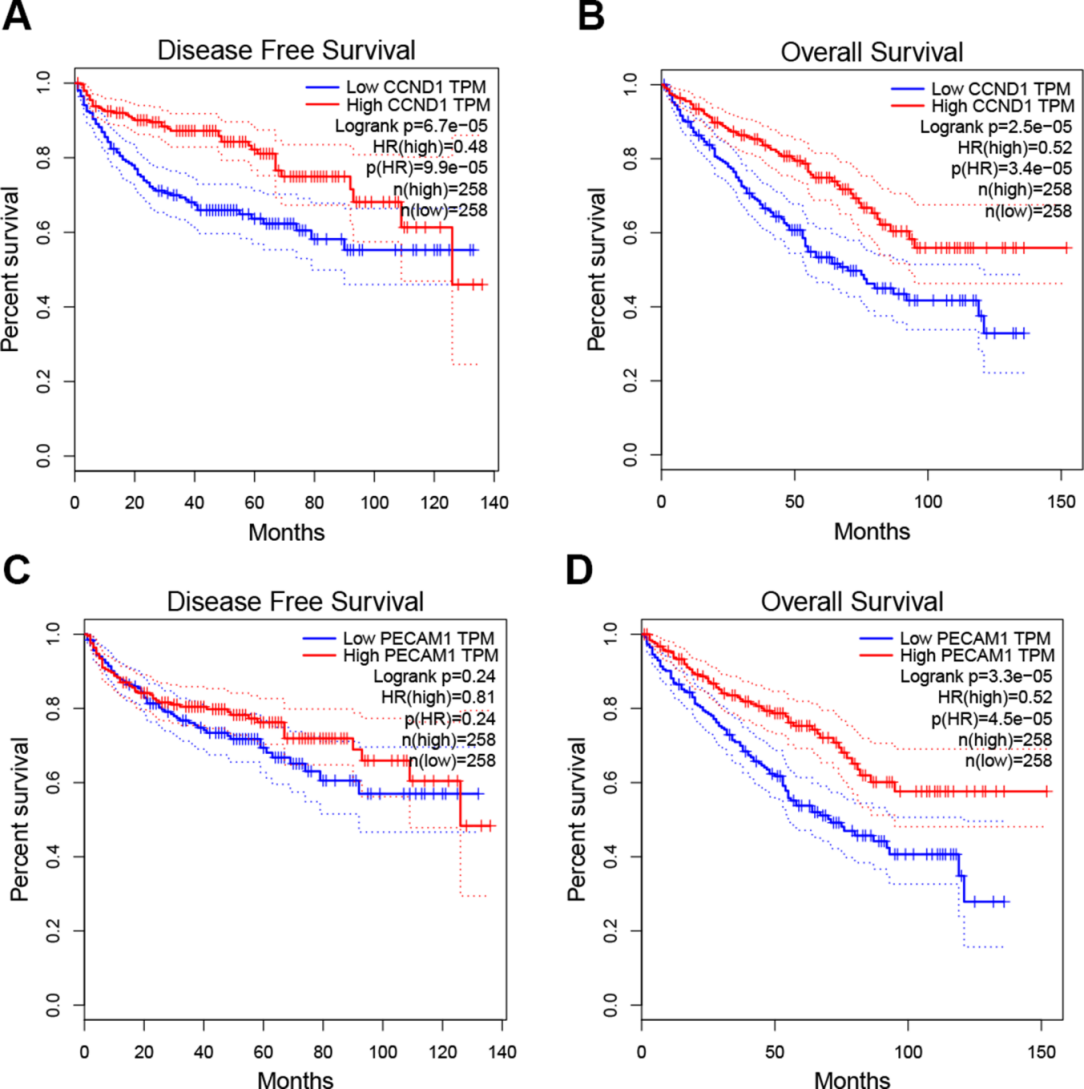
**Univariate survival analysis of the hub genes was performed using the Kaplan-Meier curve.** (**A**–**B**) The gene of CCND1 expression showed obviously significant better DFS and OS in ccRCC samples (Logrank P < .05). (**C**–**D**) The expression of the *PECAM1/CD31* gene showed a significantly better OS in the ccRCC sample, whereas there was no statistical difference in DFS. Abbreviation: DFS: disease-free survival; OS: overall survival; ccRCC: clear cell renal cell carcinoma.

To determine whether the hub genes in ccRCC have clinical relevance, we performed correlation analysis with the clinical correlative of kidney cancer outcomes in TCGA kidney cancer data sets. Using the data from gene expression profiling interactive analysis (GEPIA), we noted that ccRCC patients who had an association of genomic alterations in *CCND1* showed reductions in overall and disease-free survival (P=2.5E-05 for overall survival and P=6.7E-05 for disease-free survival) (Figure 3A–3B). In addition, the *PECAM1/CD31* alteration was significantly associated with worse overall survival (P=3.3E-05 for overall survival) while disease-free survival was not statistically significant (P=0.24 for disease-free survival) (Figure 3C–3D).

**Figure 4:**
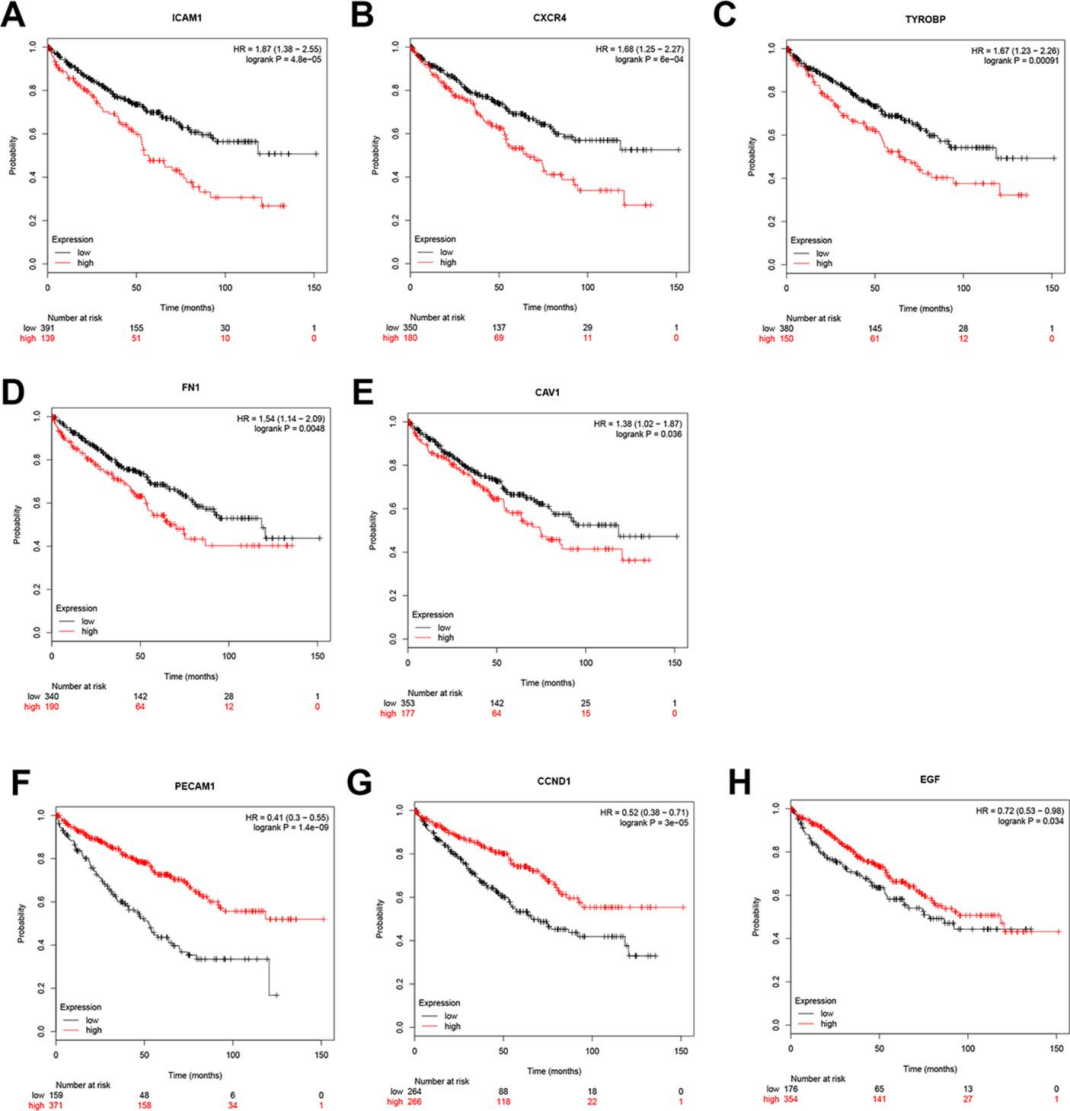
**Univariate survival analysis of the hub genes was performed using the Kaplan-Meier curve.** The 8 genes of 10 hub genes showed significant difference in OS. Each elevated expression in the 5 significant hub genes showed obviously significant worse OS in ccRCC samples, whereas elevation of the remaining 3 hub genes showed better OS (Logrank P < .05). Abbreviation: OS: overall survival; ccRCC: Clear cell renal cell carcinoma.

Additionally, when have optimized cut-off for hub gene analysis, we found that high expression of *FN1*, *ICAM1*, *CXCR4*, *TYROBP*, *EGF*, and *CAV1* are associated with poor prognosis (Figure 4) and suggested that these genes can also be used as indicators to monitor prognosis.

### Differential expression of CCND1 and PECAM1/CD31

**Figure 5:**
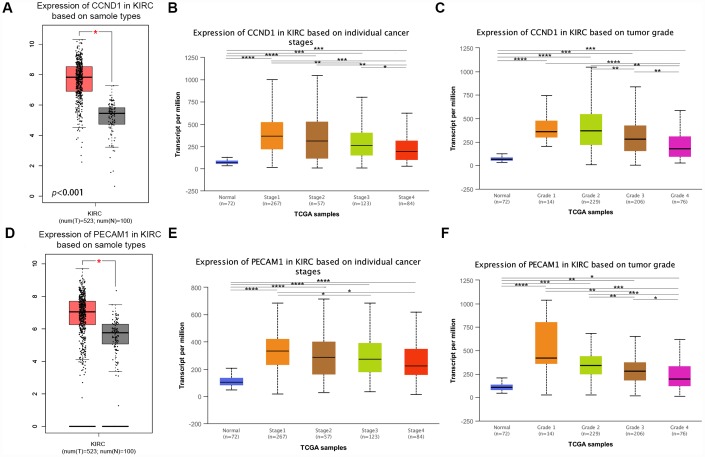
**Transcriptional expression of *CCND1* and *PECAM1/CD31* in ccRCC tumor tissues and adjacent normal renal tissues.** (**A**) Transcriptional level of *CCND1* expression was found highly expressed in 533 ccRCC tissues compared with 72 normal tissues (*p*<0.0001). (**B**) Transcriptional expression of *CCND1* was significantly correlated with AJCC stages, patients who were in more mild stages tended to express higher mRNA expression of *CCND1*. (**C**) Transcriptional expression of *CCND1* was significantly correlated with ISUP grade, patients who were in more mild grade score tended to express elevated mRNA exspression of *CCND1*. Highest mRNA expressions of *CCND1* were found in stage 1 or grade 1. (**D**) Transcriptional level of *PECAM1/CD31* expression was found highly expressed in 533 ccRCC tissues compared with 72 normal tissues (*p*<0.0001). (**E**) Transcriptional expression of *PECAM1/CD31* was significantly correlated with AJCC stages, patients who were in more mild stages tended to express higher mRNA expression of *PECAM1/CD31*. (**F**) Transcriptional expression of *PECAM1/CD31* was significantly correlated with ISUP grade, patients who were in more mild grade score tended to express elevated mRNA expression of *PECAM1/CD31*. Highest mRNA expressions of *PECAM1/CD31* were found in stage 1 or grade 1.

The mRNA expression of *CCND1* and *PECAM1/CD31* were compared between kidney tumor samples and adjacent normal tissues respectively based on RNA-sequence data from TCGA database. Transcriptional level of *CCND1* expressions were found highly expressed in 533 ccRCC tissues compared with 72 normal tissues (Figure 5A). As was shown in Figure 5B, *CCND1* mRNA expressions of ccRCC samples were significantly correlated with mild clinical stages, and the highest *CCND1* mRNA expressions were found in stage 1. Similarly, relationship between *CCND1* mRNA expression and different pathological grade was measured, which suggested that mRNA expressions of *CCND1* were significantly correlated with pathological grades (Figure 5C). In addition, mRNA level of *PECAM1/CD31* was also increased in ccRCC tissues (Figure 5D). *PECAM1/CD31* mRNA expression in the ccRCC sample was also significantly correlated with mild clinical staging, and the highest *PECAM1/CD31* mRNA expression was found in stage 1 (Figure 5E). Meanwhile, mRNA expression levels of *PECAM1/CD31* were also associated with lower clinicopathological grading (Figure 5F).

In addition, representative proteins expressions of immunohistochemistry images indicated that *CCND1* staining was not detected in normal kidney tissues, while its medium staining was observed in ccRCC tissues (Supplementary Figure 1). Taken together, it suggested that transcriptional and proteomic expressions of *CCND1* were highly expressed in ccRCC tissues compared with normal tissues. Therefore, the hub gene *CCND1* and *PECAM1/CD31* may play key role in the progression of clear cell renal cell carcinoma. Overall, elevated expression of *CCND1* and *PECAM1/CD31* mRNA was significantly associated with mild clinical pathological parameters in ccRCC patients and was only significantly elevated in the early stages of the disease. Therefore, it may play an important role in the early diagnosis of ccRCC.

### Clinicopathological characteristics baseline for ccRCC patients and cox regression analyses of CCND1 as well as PECAM1/CD31 in TCGA cohort

This study included 533 selected samples in TCGA cohort. As shown in Supplementary Table 1, the clinical characteristics of these samples contained information regarding age, gender, laterality, T stage, N stage, M stage AJCC stage, and ISUP grade.

Univariate and multivariate analyses were conducted to identify OS-correlated characteristics. In univariate Cox regression analysis models of *CCND1*, traditional prognostic factors such as pTNM stage, AJCC stage, and ISUP grade were significantly relevant to OS (*p*<0.05; Supplementary Table 2) in ccRCC patients in the TCGA cohorts. Importantly, *CCND1* amplification markedly correlated with poor OS (hazard ratio [HR]=0.408, *p*<0.001). In multivariate Cox regression analysis, traditional prognostic factors, specifically pM stage, were still relevant to OS (HR=2.764, *p*<0.001; Supplementary Table 2) in ccRCC patients. Importantly, elevated *CCND1* expression was significantly associated with poor OS (HR=0.603, *p*=0.043) in TCGA cohorts of ccRCC patients.

In addition, in univariate Cox regression analysis models of *PECAM1/CD31*, traditional prognostic factors such as pTNM stage, AJCC stage, and ISUP grade also were significantly relevant to OS (*p*<0.05; Supplementary Table 3) in ccRCC patients in the TCGA cohorts. Significantly, *PECAM1/CD31* amplification markedly correlated with poor OS (HR=0.448, *p*<0.001). In addition, in multivariate Cox regression analysis, traditional prognostic factors such as pM stage, were still relevant to OS (HR=2.971, *p*<0.001; Supplementary Table 3) in ccRCC patients. Importantly, elevated *PECAM1/CD31* expression was significantly associated with poor OS (HR=0.595, *p*=0.016) in TCGA cohorts of ccRCC patients.

### External prognostic validation of CCND1 and PECAM1/CD31

**Figure 6:**
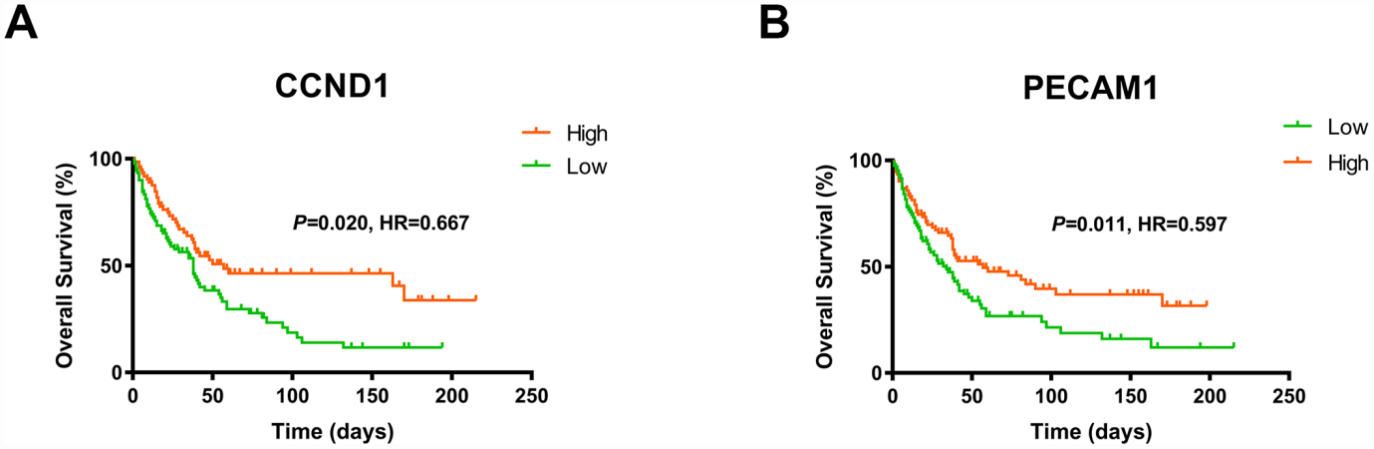
**The gene of *CCND1* and *PECAM1/CD31* expression significantly correlated OS and PFS in an independent external ccRCC cohort GSE3538.**

Consistent with previous results, we enrolled survival and follow-up data from an independent cohort GSE3538 (Zhao Renal dataset). It suggested that high expression of *CCND1* and *PECAM1/CD31* in GSE3538 were significantly associated with favorable prognosis in ccRCC patients (Figure 6) [9].

**Figure 7:**
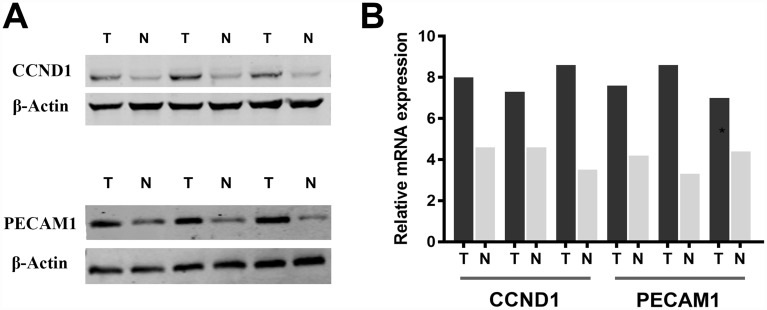
Significantly elevated CCND1 and PECAM1/CD31 expression in human ccRCC tissues compared with normal tissues in (**A**) protein and (**B**) mRNA levels.

Subsequently, we measure CCND1 and PECAM1/CD31 expression level in three pairs of ccRCC tumor and normal samples. Significantly elevated CCND1 and PECAM1/CD31 expression in human ccRCC tissues compared with normal tissues in protein and mRNA levels (Figure 7A–7B).

### Expression of CCND1 and PECAM1 other types of tumors and prognostic value

We have already discussed the better prognosis of ccRCC patients with high expression of *CCND1* and *PECAM1/CD31*. To assess the expression and prognostic value of *CCND1* and *PECAM1/CD31* in other tumors, we used the tumor-immune system interactions (TISIDB, http://cis.hku.hk/TISIDB/index.php) online database to detect the expression of *CCND1* and *PECAM1/CD31* in other types of tumors other than kidney cancer and to assess the prognostic value based on TCGA cohort. As shown in Supplementary Figure 2, the high expression of *CCND1* is not only related to the better prognosis of patients with kidney renal clear cell carcinoma, but also related to the better prognosis of liver hepatocellular carcinoma. However, the high expression of CCND1 is associated with poor prognosis of head and neck squamous cell carcinoma, lung adenocarcinoma, mesothelioma and pancreatic adenocarcinoma. In addition, while the high expression of *PECAM1/CD31* is related to the better prognosis of patients with kidney renal clear cell carcinoma, and skin cutaneous melanoma, the high expression of *PECAM1/CD31* is related to the poor prognosis of patients with brain low grade glioma and uveal melanoma(Supplementary Figure 3).

### GSEA analysis

**Figure 8:**
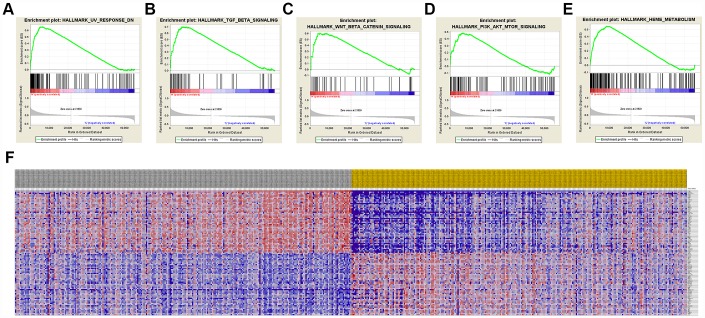
**Significant CCND1-related genes and hallmarks pathways in ccRCC obtained by GSEA.** A total of 100 significant genes were obtained by GSEA with positive and negative correlation. (**A**–**E**) The most involved significant pathways included hedgehog signaling, heme metabolism, PI3K/AKT/MTOOR signaling, TGF-β signaling, UV-response, and WNT/β/CATEMIN signaling. (**F**) Transcriptional expression profiles of the 100 significant genes were performed in a heat map.

**Figure 9:**
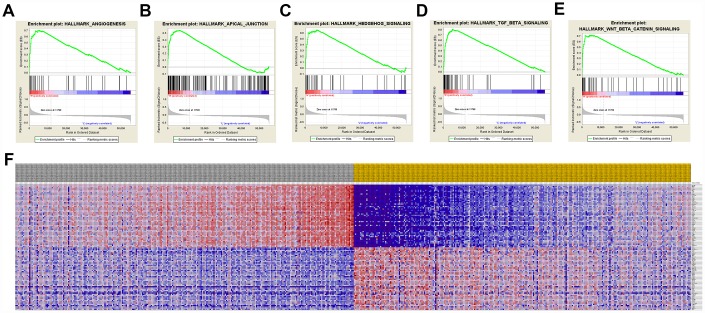
**Significant PECAM1/CD31-related genes and hallmarks pathways in ccRCC obtained by GSEA.** A total of 100 significant genes were obtained by GSEA with positive and negative correlation. (**A**–**E**) The most involved significant pathways included angiogenesis, apical junction, hedgehog signaling, TGF-beta signaling, and WNT/β/CATEMIN signaling. (**F**) Transcriptional expression profiles of the 100 significant genes were performed in a heat map.

A total of 100 significant genes were obtained by GSEA with positive and negative correlation. Importantly, GSEA was used to perform hallmark analysis for *CCND1* and *PECAM1/CD31*. Results suggested that the most involved significant pathways of *CCND1* included hedgehog signaling, heme metabolism, PI3K/AKT/mTOR signaling, TGF-β signaling, UV-response, and wnt-β/catemin signaling. The details are shown in Figure 8A–8E. In addition, transcriptional expression profiles of the 100 significant genes were performed in a heat map (Figure 8F). In addition, GSEA enrichment analysis showed that the most important pathways for *PECAM1/CD31* include angiogenesis, apical junction, hedgehog signaling, TGF-beta signaling, and WNT/β/CATEMIN signaling. The details are shown in Figure 9A–9E. Meanwhile, transcriptional expression profiles of 100 significant genes related to *PECAM1/CD31* were performed in the heat map (Figure 9F).

## DISCUSSION

Clear cell renal cell carcinoma is one of the most common genitourinary system malignancies in the world. It is well known that advanced ccRCC is difficult to treat while early ccRCC has a favorable prognosis. Although various causes and underlying mechanisms of ccRCC formation and progression were investigated in multiple “omics” studies, the global morbidity and mortality of ccRCC has remained high over the past few decades. Similar to other solid tumors, the development and progression of ccRCC is characterized by abnormal genetic and protein expression. Numerous studies have demonstrated that development of ccRCC is the result of an accumulation of cellular and molecular aberrations, including epigenetic, transcriptomic, miRNA, proteomic and metabolomic abnormalities [10–13]. The multiple “omics” research that aim to identify diagnostic biomarkers for early detection of kidney cancer, highlight not only the heterogeneity, but also the potential molecular commonalities in different stages of kidney cancer. Clearly, ccRCC has significant molecular heterogeneity, involving numerous genetic and protein level changes, thus, a group of well-selected candidates could represent all of these tumors. With the development of bioinformatics, several molecular markers in ccRCC have been identified as potential novel prognostic biomarkers, but none of them has been independently validated. Neither have these biomarkers been compared with each other to determine further studies and screen candidates. The molecular signature of ccRCC, which may provide potential biomarkers for early detection and monitoring for progression, is still far from fully defined.

In our study, unlike a single genetic or cohort study, the study analyzed three microarray datasets, including 63 kidney tumors and 54 normal kidney samples. A total of 379 DEGs were identified in ccRCC, including 249 upregulated genes and 130 downregulated genes. Gene ontology was performed based on DAVID, which showed that the DEGs were mainly enriched in angiogenesis, extracellular matrix organization, response to hypoxia, excretion and response to drug categories. Pathway enrichment was analyzed based on the KEGG database to evaluate the functional relevance of DEGs. Based on the STRING database and Cytoscape software, relevant PPIs were constructed and visualized that contained 379 nodes and 1704 edges.

The most significant 10 hub genes were selected, and comprised *VEGFA*, *FN1*, *ITGB2*, *ICAM1*, *CXCR4*, *PECAM1/CD31*, *TYROBP*, *CCND1*, *EGF*, and *CAV1*. To screen for early diagnosis of renal cancer, we selected two genes that were elevated in the early stages of renal cell carcinoma: *CCND1* and *PECAM1/CD31*.

The gene *CCND1* encodes the cyclin D1 protein, a member of the cyclin family involved in the regulation of cell cycle progression. Cyclin D1, dimerizes with cyclin-dependent kinase (CDK) 4 or CDK6 to regulate the G1/S phase transition of the cell cycle [14]. Previous studies have showed that cyclin D1 plays a vital role in tumors of the breast [15], lung [16], bladder [17] and colon [18]. In a recent study, He et al. observed that expression of cyclin D1 in breast cancer tissue was higher than in normal samples, and the expression levels were significantly correlated with tumor size, clinical stage and pathological grade [19]. The relationship between *CCND1* and renal cell carcinoma has not yet been fully determined, but its tendency to increase expression in the early stages of renal cell carcinoma may serve as an indicator for early diagnosis of renal cell carcinoma.

Platelet and endothelial cell adhesion molecule 1, (*PECAM1,* also named *CD31*), encodes a protein involved in several processes of primary tumor growth and proliferation, including angiogenesis, vascular permeability, and extracellular circulation [20]. There is evidence that *PECAM1/CD31* is involved in the progression of a variety of malignancies including melanoma and cancers of the lung and breast [21, 22]. There is a need to further explore the molecular mechanisms between the expression of *PECAM1/CD31* and tumorigenesis, and identify potential therapeutic targets. Like *CCND1*, *PECAM1/CD31* was also observed to be highly expressed in the early stages of ccRCC. This suggests that *PECAM1/CD31* and *CCND1* could be used as indicators for the early diagnosis of renal cancer.

Gene set enrichment analysis (GSEA) showed that significant pathways for *CCND1* and *PECAM1/CD31* include the transforming growth factor (TGF)-β and Wnt pathways. TGF-β signaling events are well known to control diverse processes and numerous responses, such as cell proliferation, differentiation, apoptosis, and migration [23]. In the early stages of tumor development, TGF-β often acts as a tumor suppressor, whereas in later phases, cancer cells can become resistant to its antimitogenic effects and TGF-β can shift into a tumor promoter [24–26]. In the later phases of cancer progression, TGF-β signaling can reduce expression of epithelial markers, such as E-cadherin, and promote epithelial-to-mesenchymal transition (EMT) by increasing the expression of mesenchymal markers including N-cadherin and vimentin [27, 28]. EMT is essential for normal embryonic development, but it also contributes to tumor invasion and metastasis. Recent studies have confirmed that TGF-β is involved in bone metastasis of breast cancer and prostate cancer [29–31]. Because TGF-β has a wide range of functions during tumor metastasis, specific blocking of TGF-β ligand and/or receptor activity may provide better antitumor therapeutic effects. Several clinical trials of drugs that target TGF-β are currently underway including those that are aimed at cancer progression and metastasis [32].

The Wnt signaling pathway has also been intensively studied and plays a role in cell proliferation, growth, cell fate and differentiation [33, 34]. Mutations in Wnt signaling pathway components are linked to many diseases, including cancer [35]. It is important to understand that the Wnt signaling pathway also provides potential benefits for genetic therapy. The components of the Wnt signaling pathway can be divided into Wnt ligands and Wnt receptors [36]. There are 19 Wnt ligands, all of which have a cysteine-rich domain and can activate different types of Wnt signaling pathways by binding to specific Wnt signaling receptors [37]. In addition, some Wnt ligands are also involved in the formation and development of cancer. *WNT1* encodes a number of glycoproteins that are reported to be markers of advanced metastasis in cancer patients [38]. WNT3A was found to be overexpressed and correlated with the level of MMP9 in colorectal tumor tissues [39]. Wnt signaling is involved in determining cell fate, and mutations in Wnt signaling pathway components are reported to be strongly associated with different types of human cancers, such as lung, breast and ovarian cancer [40–42]. Thus, inhibitors of Wnt signaling could be used for cancer treatment.

The phosphatidylinositol 3-kinase (PI3K) signaling pathway is overactivated in most cancers [43]. PI3K signaling plays an important role in cellular physiology, coordinates insulin signaling during organism growth, and mediates key cellular processes such as glucose homeostasis, protein synthesis, cell proliferation and survival. This pathway has been a fierce field of investigation, especially in light of cancer genome studies that have showed it is one of the most common pathways altered in human malignancies. PI3K signaling impacts on many processes that regulate the hallmarks of cancer, including cell proliferation, survival, and genomic instability, and metabolism [44].

Our study is an attempt to construct a gene regulatory network incorporating DEGs that have been identified in normal tissues and primary ccRCC, and to initiate the functional annotation of hub genes that they share. Furthermore, we believe that alterations in *ICAM1*, *CXCR4*, *TYROBP*, *FN1*, and *CAV1* are significantly associated with worse prognosis, indicating that these genes may play important roles in creating aggressive malignant phenotypes of ccRCC. This study had the following limitations. First, the data we utilized were drawn from unbalanced samples of normal tissues and primary ccRCCs, which were of poor quantity and downloaded from the GEO database, rather than samples obtained by our team. Second, this study failed to explore underlying mechanisms of signaling pathways in ccRCC, although a series of functional annotations and enrichment analyses were investigated. Further research should focus on exploring the detailed mechanisms between the hub genes and ccRCC carcinogenesis.

In conclusion, this study has identified DEGs and hub genes in normal tissues and primary ccRCC tissues, that may help us uncover the mechanisms behind the development of ccRCC, and provide more clues for its prognosis. Further research is needed to elucidate the molecular pathogenesis and alteration of the signaling pathways of the hub genes in ccRCC.

## MATERIALS AND METHODS

### Transcriptional expression profiles process

NCBI-GEO is regarded as a free public database of transcriptional expression profile and we obtained the gene expression profile of GSE15641, GSE16441 and GSE66270 in kidney cancer and normal kidney tissues. The data of GSE15641 were obtained with the GPL96 platform (Affymetrix Human Genome U133A Array) and came from 32 kidney tumours and 23 normal kidney tissue sample. Similarly, the data of GSE16441 were based on the GPL6480 platform (Whole Human Genome Microarray 4x44K G4112F). The gene microarray data were collected from 17 renal clear cell carcinoma and 17 non-tumor. The GSE66270 data were obtained from the GPL570 platform (Affymetrix Human Genome U133 Plus 2.0 Array). 14 primary kidney tumours and 14 Adjacent Normal kieney tissues were analysed for the GSE66270 data set.

### Standardization and elucidation of DEGs

DNA microarray analysis started with preprocessing and standardization of raw biological data. This procedure eliminated noise from the biological data and guaranteed integrity. Then, background correction of probe data, standardization, and summarization were performed by robust multiarray average analysis algorithm [45] in affy package of R [46].

The DEGs between normal kidney tissues and ccRCC samples were screened and identified across experimental conditions. Delineating parameters such as adjusted P values (adj. P), Benjamini and Hochberg false discovery rate (FDR) and fold change were utilized to filter DEGs and provide a balance between the discovery of statistically significant genes and limitations of falsepositives. Probe sets without corresponding gene symbols or genes with more than one probe set were removed or averaged. The absolute value of |log2FC| (fold change) >1.00 and P value <.05 were considered statistically significant. Then, the raw data in TXT format were checked in Venn software online to detect the commonly DEGs among the three datasets. The DEGs with log FC < 0 was considered as down-regulated genes, while the DEGs with log FC > 0 was considered as an up-regulated gene.

### KEGG and GO enrichment analyses of DEGs

Database for Annotation, Visualization and Integrated Discovery (DAVID) (http://david.ncifcrf.gov) (version 6.7) is an online biological information database that integrates biological data and analysis tools, and provides a comprehensive set of functional annotation information of genes and proteins for users to extract biological information [47]. Kyoto Encyclopedia of Gene and Genome (KEGG) is a database resource for understanding advanced functions and biological systems from large-scale molecular data generated by high-throughput experimental techniques [48]. Gene ontology (GO) is a major bioinformatics tool for annotating genes and analyzing the biological processes of these genes [49]. We could use DAVID to visualize the DEGs enrichment of molecular function (MF), cellular components (CC), biological processes (BP) and biological pathways (P < 0.05).

### Human protein atlas

The Human Pathology Atlas project (https://www. proteinatlas.org) contains immunohistochemistry (IHC) data using a tissue microarray-based analysis [50]. Staining intensity, quantity, location and patients’ information in patients with the respective cancer types were available online. In this study, representative proteins expressions of IHC images of CCND1 were detected in ccRCC and normal tissues in Human Protein Atlas.

### PPI network and module analysis

DEG-encoded proteins and the PPI were obtained using the online database STRING (available online: http://string-db.org) [51]. An interaction with a combined score >0.4 was considered statistically significant. Cytoscape (version 3.7.1), a public source bioinformatics software platform, was used to visualize and analyze molecular interaction networks [52]. The plug-in Molecular Complex Detection (MCODE) (version 1.5.1) of Cytoscape was used for clustering a given network based on the topology to find densely connected regions [53]. MCODE can be used to identify the most dense and significant module in the PPI networks with criteria as follows: degree cut-off = 2, node score cut-off = 0.2, Max depth = 100, and K-score = 2.

### Hub genes selection and analysis

The plug-in cytoHubba (version 0.1) of Cytoscape was used for screen out hub genes based on degress. The hub genes were selected with degrees ≥10(each node had more than 11 connections/interactions). A network of the genes and their co-expression genes was analyzed using cBioPortal (http://www.cbioportal.org) online platform [54]. ClueGO is a plug-in of Cytoscape that can visualize the nonredundant biological terms for large clusters of genes in a functionally grouped network [55]. The biological process from GO analysis of hub genes was performed and visualized by ClueGO (version 2.5.4) and CluePedia (version 1.5.4), which was a functional extension of ClueGO and a plug-in of Cytoscape [56]. In addition, hierarchical clustering of the hub genes was constructed.

### Survival analysis

The Kaplan-Meier method was used to analyze differences in survival between groups. The primary endpoint was disease-free survival (DFS), the duration from the onset of curative treatment to the date of progression or the start date of the second-line treatment or the date of death, whichever occurs first. The overall survival (OS) as a secondary endpoint was the length of time from the date of diagnosis or first treatment to the date of death or last follow-up. The Kaplan-Meier method was used to estimate and account for the duration of follow-up, the 95% confidence interval (95% CI) and the log-rank test in the separation curve. The overall score was determined as the sum of the weights of each important central gene. Univariate and multivariate analysis were performed with Cox logistic regression models to find independent variables, including age at diagnosis, age (ref. < 60 years), gender (ref. Male), pT stage (ref. T1-T2), pN stage (ref. N0), pM stage (ref. M0), AJCC stage (ref. I-II), ISUP grade (ref. 1-2) and CCND1 and PECAM1/CD31 expression (ref. Low). X-tile software was utilized to take the cut-off value. All hypothetical tests were two-sided and p-values less than 0.05 were considered significant in all tests. Integrated score was identified as sum of the weight of CCND1 and PECAM1/CD31 and significant clinicopathological prognostic indicators.

### Oncomine database

In this study, transcriptional expression profiles of *CCND1* and *PECAM1/CD31* in ccRCC patients were obtained from Oncomine database using Oncomine online database (http://www.oncomine.com) [57]. Difference of transcriptional expression was compared by Students’t-test. Cut-off of *p* value and fold change were as following: *p*-value=0.01, Fold Change=1.5, gene rank=10%, Data type: mRNA.

### Western blotting analysis

Total protein was extracted from cells using RIPA lysis buffer (TaKaRa) according to the manufacturer’s instructions. Proteins in lysates were determined using the bicinchoninic acid (BCA) assay and 10% SDS-PAGE and then transferred onto a polyvinylidene fluoride (PVDF) membrane. The membrane was incubated with blocking buffer for 2 h at room temperature and then with the primary antibody anti-CCND1 (1:1000, ab16663, Abcam) and and anti-CD31 (PECAM1/CD31; 1:1000, ab28364, Abcam) overnight at 4°C. Then, the protein was visualized using ECL plus western blotting detection reagents (Biosciences) and detected with an enhanced chemiluminescence kit.

### RNA extraction and quantitative real-time PCR analysis (qRT-PCR)

Total RNA from harvested patients’ sample cells was isolated by Trizol (Invitrogen, Carlsbad, CA), and qRT-PCR was performed using SYBR® Premix Ex TaqTM (TaKaRa) according to manufacturer’s protocol, as previously described [58]. The primers pairs were: CCND1 forward, 5′-GCTGTGCATCTACACCGACA-3′, CCND1 reverse, 5′-TTGAGCTTGTTCACCAGGAG-3′, CD31/PECAM1/CD31 forward, 5′-GTGCTGCAATGTGCTGTGAA-3′, CD31/PECAM1/CD31 reverse, 5′-CTGGTTCGTCTTCCGATCTGT-3′. The mRNA expression level was normalized to actin and replicated in triplicate according to the manufacturer’s instructions. The relative expression quantity was calculated using the 2^-ΔΔCt^ method.

### Data processing of gene set enrichment analysis (GSEA)

TCGA database were implemented with GSEA method using the Category version 2.10.1 package. Student’s-t-test statistical score was performed in consistent pathways and the mean of the differential expression genes was calculated. A permutation test with 1000 times was used to identify the significantly changed pathways. The adjusted P values (adj. P) using Benjamini and Hochberg (BH) false discovery rate (FDR) method by default were applied to correct the occurrence of false positive results [23]. The significant related genes were defined with an adj. P less than 0.01 and FDR less than 0.25. Statistical analysis and graphical plotting were conducted using R software (Version 3.6.1).

### Ethics approval

The Ethics Committee of Longhua Hospital Affiliated to Shanghai University of Traditional Chinese Medicine approved the study.

## Supplementary Material

Supplementary Figures

Supplementary Tables
