# Supplementary material for: Screening, identification and validation of CCND1 and PECAM1/CD31 for predicting prognosis in renal cell carcinoma patients
Source: Aging (Albany NY). 2019 Dec 18;11(24):12057–79. doi: 10.18632/aging.102540 (PMC6949065; doi:10.18632/aging.102540)
Supplement: Supplementary Figures [file aging-11-102540-s002..pdf]

SUPPLEMENTARY FIGURES

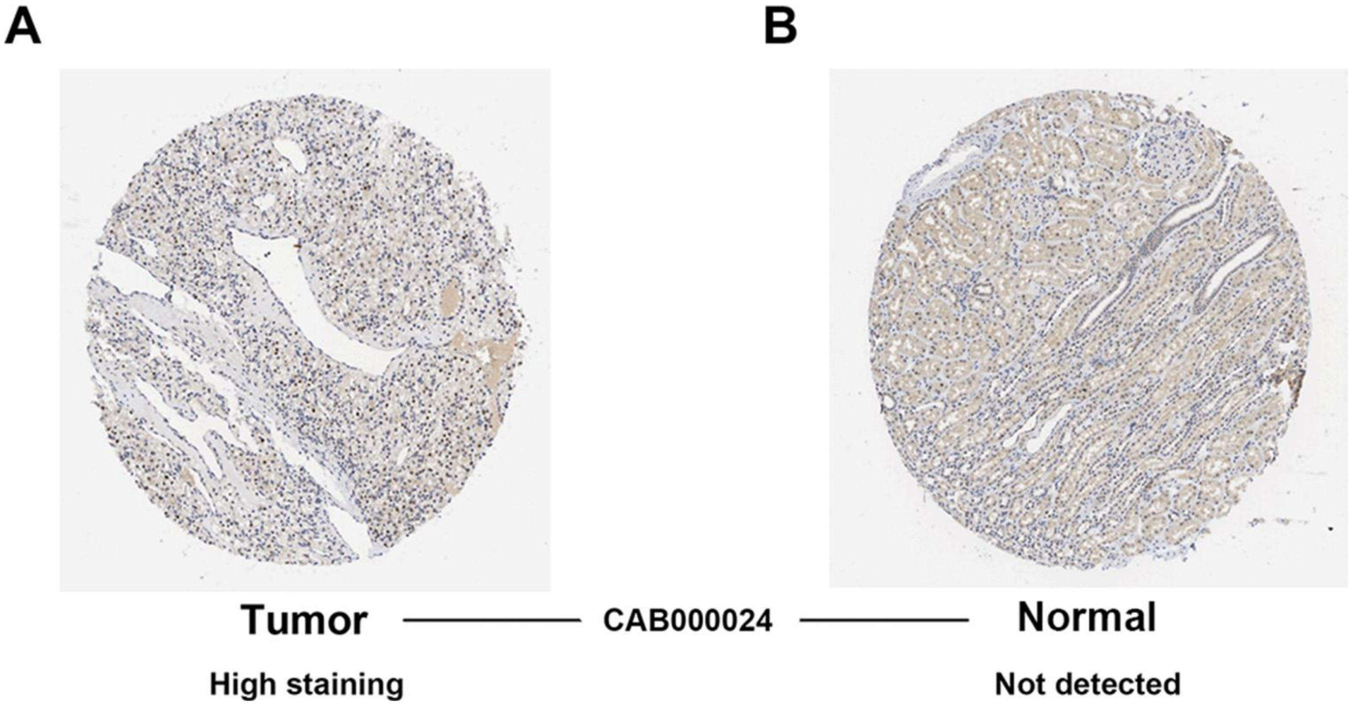

Supplementary Figure 1. ***CCND1*** **proteins expression significantly high staining expressions were observed in ccRCC tissues.**

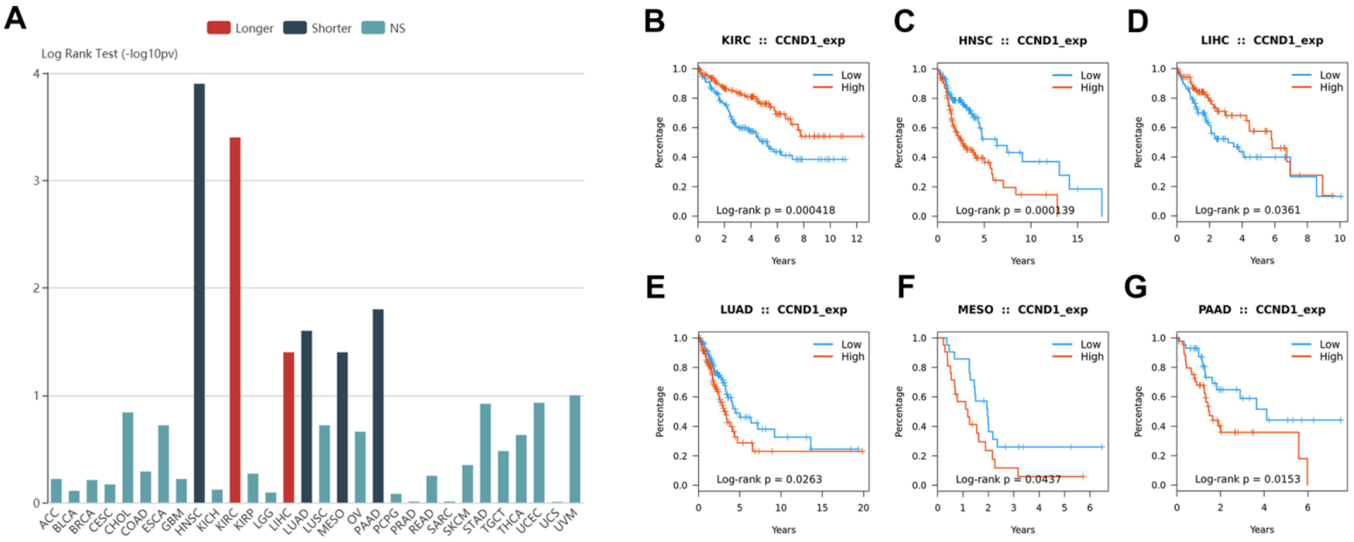

Supplementary Figure 2. (**A**) The expression and prognostic value of CCND1 in tumors based on TCGA cohort. (**B**–**G**)High expression of CCND1 is related to the better prognosis of patients with kidney renal clear cell carcinoma and liver hepatocellular carcinoma. However, high expression of CCND1 is associated with poor prognosis of head and neck squamous cell carcinoma, lung adenocarcinoma, mesothelioma and pancreatic adenocarcinoma.

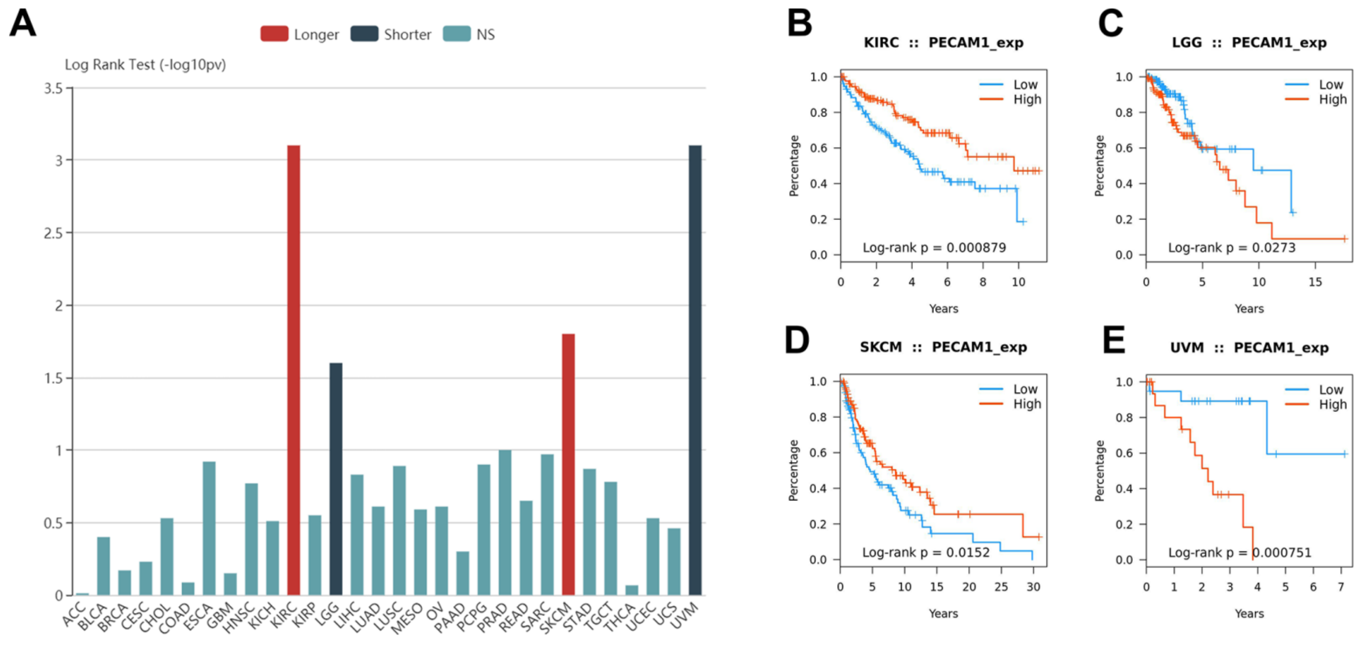

Supplementary Figure 3. (**A**) The expression and prognostic value of PECAM1/CD31 in tumors based on TCGA cohort. (**B**–**E**) The high expression of PECAM1/CD31 is related to the better prognosis of patients with kidney renal clear cell carcinoma and skin cutaneous melanoma However, the high expression of PECAM1/CD31 is related to the poor prognosis of patients with brain low grade glioma and uveal melanoma.
