# Supplementary material for: Screening, identification and validation of CCND1 and PECAM1/CD31 for predicting prognosis in renal cell carcinoma patients
Source: Aging (Albany NY). 2019 Dec 18;11(24):12057–79. doi: 10.18632/aging.102540 (PMC6949065; doi:10.18632/aging.102540)
Supplement: Supplementary Tables [file aging-11-102540-s001..pdf]

SUPPLEMENTARY TABLES

Supplementary Table 1. Clinicopathological characteristics baseline for ccRCC patients in TCGA cohort. (ccRCC: clear cell renal cell carcinoma; TCGA: The Cancer Genome Atlas)

Characteristics	TCGA cohort (N=533)
N (%)	
Age	
< 60 years	245 (46.0)
≥ 60 years	288 (54.0)
Gender	
Male	345 (64.7)
Female	188 (35.3)
Laterality	
Left	251 (47.1)
Right	282 (52.9)
pT stage	
T1	273 (51.2)
T2	69 (12.9)
T3	180 (33.8)
T4	11 (2.1)
pN stage	
N0	240 (45.0)
N1	16 (3.0)
Nx	277 (52.0)
pM stage	
M0	422 (79.2)
M1	79 (14.8)
Mx	32 (6.0)
AJCC stage †	
I	267 (50.1)
II	57 (10.7)
III	123 (23.1)
IV	86 (16.1)
ISUP grade	
G1	14 (2.6)
G2	229 (43.0)
G3	206 (38.6)
G4	76 (14.3)
Gx	8 (1.5)

Supplementary Table 2. Univariate Cox logistic regression analysis of CCND1 for predicting OS in TCGA cohort (OS: overall survival; TCGA: The Cancer Genome Atlas)

Covariates	Univariate analysis			Multivariate analysis		
	HR	95% CI	*P* value	HR	95% CI	*P* value
Age	1.795	1.311-2.456	**<0.001**	1.028	1.010-1.047	**0.002**
Gender (ref. Male)	1.054	0.775-1.434	0.737	-	-	-
pT stage (ref. T1-T2)	3.138	2.320-4.245	**<0.001**	1.520	0.675-3.421	0.312
pN stage (ref. N0)	3.380	1.795-6.367	**<0.001**	1.703	0.852-3.405	0.132
pM stage (ref. M0)	3.589	2.636-4.886	**<0.001**	2.764	1.654-4.620	**<0.001**
AJCC stage (ref. I-II)	3.835	2.798-5.256	**<0.001**	1.153	0.453-2.933	0.765
ISUP grade (ref. G1-G2)	2.651	1.887-3.723	**<0.001**	1.504	0.920-2.460	0.104
CCND1 expression (ref. low)	0.408	0.297-0.561	**<0.001**	0.603	0.369-0.985	**0.043**

Supplementary Table 3. Univariate Cox logistic regression analysis of PECAM1/CD31 for predicting OS in TCGA cohort (OS: overall survival; TCGA: The Cancer Genome Atlas)

Covariates	Univariate analysis			Multivariate analysis		
	HR	95% CI	*P* value	HR	95% CI	*P* value
Age	1.795	1.311-2.456	**<0.001**	1.031	1.012-1.050	**<0.001**
Gender (ref. Male)	1.054	0.775-1.434	0.737	-	-	-
pT stage (ref. T1-T2)	3.138	2.320-4.245	**<0.001**	1.662	0.734-3.767	0.223
pN stage (ref. N0)	3.380	1.795-6.367	**<0.001**	1.919	0.964-3.818	0.064
pM stage (ref. M0)	3.589	2.636-4.886	**<0.001**	2.971	1.773-4.978	**<0.001**
AJCC stage (ref. I-II)	3.835	2.798-5.256	**<0.001**	1.170	0.460-2.972	0.742
ISUP grade (ref. G1-G2)	2.651	1.887-3.723	**<0.001**	1.484	0.906-2.429	0.117
PECAM1 expression (ref. low)	0.448	0.333-0.603	**<0.001**	0.595	0.390-0.906	**0.016**
